# A Novel Ice Pillow Cooling Technique Reduces Renal Rewarming During Simulated Vascular Anastomosis: An Ex Vivo Study Using Human Renal Allografts

**DOI:** 10.3390/jcm15156001

**Published:** 2026-08-02

**Authors:** Martin Igbokwe, Saeed Farzamfar, Khushi Vyas, Cory Byrne, Larry Jiang, Ali Bozaci, Alp Sener, Patrick P. Luke

**Affiliations:** 1 Multi-Organ Transplant Program, London Health Sciences Centre, London, ON N6A 5A5, Canada; martin.igbokwe@lhsc.on.ca (M.I.); ali.bozaci@lhsc.on.ca (A.B.); alp.sener@lhsc.on.ca (A.S.); 2 Matthew Mailing Center for Translational Transplant Studies, London Health Sciences Centre, London, ON N6A 5A5, Canada; saeed.farzamfar@lhsc.on.ca (S.F.); larry.jiang@lhsc.on.ca (L.J.); 3 Schulich School of Medicine and Dentistry, London, ON N6A 5A5, Canada; kvyas27@uwo.ca (K.V.); cbyrne2027@meds.uwo.ca (C.B.); 4 Department of Surgery, Division of Urology, London Health Sciences Centre, University of Western Ontario, London, ON N6A 5A5, Canada

**Keywords:** kidney transplantation, renal hypothermia, warm ischemia, ischemia–reperfusion injury, graft preservation, intraoperative cooling, renal allograft, vascular anastomosis, organ preservation, transplant surgery, ex vivo

## Abstract

**Background**: Preservation of renal hypothermia during vascular anastomosis remains a critical yet insufficiently optimized aspect of kidney transplantation. Rewarming during the second warm ischemic period increases metabolic activity and may exacerbate ischemia–reperfusion injury. Maintaining graft temperatures below the 15–18 °C metabolic threshold during implantation is associated with improved graft preservation, yet more than 80% of grafts exceed this threshold within 20 min under standard conditions. This study evaluated the thermal performance of a novel cooling adjunct, the Ice Pillow, in limiting renal rewarming under simulated ex vivo transplant conditions. **Methods**: Twelve human renal allografts (six donor pairs) were studied in a controlled laboratory setting following hypothermic machine perfusion. Within each donor pair, kidneys were randomly assigned to the Ice Pillow group or the Control group. Core temperature was measured using an intra-pelvic thermocouple probe, while surface temperature was assessed with a handheld infrared thermometer. Kidneys in the Ice Pillow group were positioned on a thin gauze pouch filled with ice slush. Control kidneys were rewarmed in a simulated body cavity at 30–32 °C. Temperature recordings were obtained at one-minute intervals over 40 min. Rewarming slopes were compared using donor-paired analysis. Longitudinal temperature trajectories were analyzed using a linear mixed-effects model accounting for repeated measurements within kidneys. Agreement between core and surface measurements was evaluated using Bland–Altman analysis. **Results**: The Ice Pillow reduced the rate of renal rewarming by 56.4% compared with donor-paired controls (0.261 ± 0.057 °C/min vs. 0.599 ± 0.066 °C/min; mean donor-paired difference 0.338 °C/min; 95% CI, 0.237–0.440; t(5) = 8.56, *p* < 0.001). Linear mixed-effects modelling demonstrated progressive thermal separation between groups, with Control kidneys estimated to be 4.28 °C warmer at 10 min (95% CI, 3.35–5.22; *p* < 0.001) and 10.44 °C warmer at 40 min (95% CI, 7.88–12.99; *p* < 0.001). Bland–Altman analysis of core–surface agreement demonstrated a mean bias of −0.51 °C with 95% limits of agreement of −6.46 °C to 5.43 °C, indicating that surface thermometry followed the general direction of core temperature change but demonstrated wide limits of agreement, indicating the two methods are not interchangeable. **Conclusions**: In this ex vivo model, the Ice Pillow substantially attenuated renal rewarming compared with unassisted controls. Whether this thermal effect translates into improved graft function requires prospective clinical evaluation.

## 1. Introduction

Hypothermic preservation remains a cornerstone of solid-organ transplantation. Lowering tissue temperature reduces metabolic demand, suppresses anaerobic respiration, preserves adenosine triphosphate stores, and mitigates cellular injury during ischemic periods [1]. In kidney transplantation, donor kidneys are typically preserved during transportation using hypothermic machine perfusion or static cold storage; however, maintaining optimal hypothermia during implantation—particularly throughout the second warm ischemia period—remains a practical challenge [2]. During this period, progressive rewarming increases cellular metabolic activity, accelerates depletion of energy reserves, and exacerbates ischemia–reperfusion injury [3,4]. Graft rewarming has been associated with endothelial dysfunction, oxidative stress, tubular injury, delayed graft function, and poorer early graft function, particularly when vascular anastomosis is prolonged [5,6,7,8]. Even modest increases in renal temperature during implantation may adversely affect graft viability, and strategies aimed at minimizing intraoperative rewarming have gained attention, especially in technically demanding procedures such as robotic-assisted kidney transplantation [9,10].

Existing cooling adjuncts—including ice slush jackets, phase-change materials, continuous-flow devices, and thermal barrier bags—can reduce graft temperature but are often bulky, may obscure the renal hilum, or require specialized equipment [11,12,13,14,15]. A simple, low-profile cooling method that preserves surgical access while maintaining hypothermia remains lacking.

To address this gap, we developed a simplified cooling adjunct referred to as the Ice Pillow—a thin, flat gauze pad filled with ice chips that rests on one surface of the allograft during anastomosis. This design is intended to optimize surgical visibility and maneuverability compared with other methods. The objective of this study was to evaluate the thermal performance of the Ice Pillow under controlled ex vivo conditions simulating the second warm ischemic period during kidney transplantation.

## 2. Methodology

### 2.1. Study Design and Experimental Setting

This was a prospective ex vivo study using human deceased-donor renal allografts deemed unsuitable for clinical transplantation following standard deceased-donor procurement procedures from external retrieval centers and transported under hypothermic conditions on ice. Ethical approval was obtained from the Western University Research Ethics Board (IRB No. 00000940). All deceased-donor organs used for research purposes were handled in accordance with institutional ethical standards and applicable regulatory guidelines.

Upon arrival, kidneys were maintained in a static cold condition using ice slush. Prior to the current experimental protocol, all grafts had been utilized in other experimental ex vivo machine perfusion studies. Following completion of those studies, each kidney was re-equilibrated in hypothermic solution until a target intrarenal temperature of 4–6 °C was achieved, thereby simulating the thermal state of a renal allograft immediately prior to implantation after hypothermic preservation.

**Figure 1:**
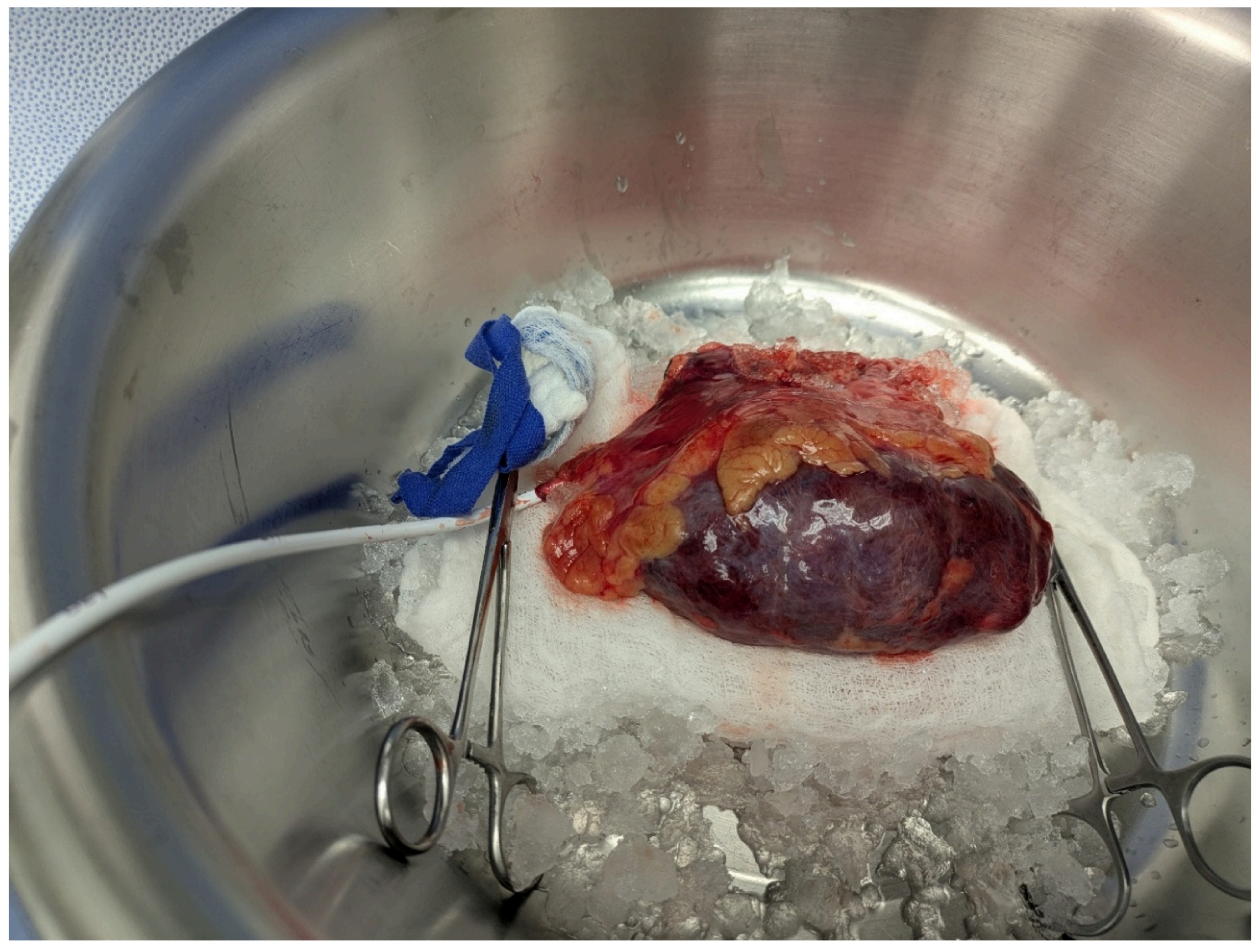
Renal allograft positioned on the Ice Pillow cooling adjunct during simulated ex vivo rewarming.

A total of twelve renal allografts (six pairs of kidneys) were included. Within each donor pair, the right and left kidneys were assigned to Ice Pillow or Control using a sealed opaque envelope method. Envelopes were prepared in advance by a team member not involved in the experiment and opened immediately before each experiment. Kidneys were assigned in a 1:1 fashion into one of two experimental groups:

1. Control (in warm cavity) Group—graft rested directly on the fluid-filled basin covered with an isolation bag and temperature maintained at 30–32 °C to simulate the temperature of the recipient pelvic side wall during vascular anastomosis without active cooling support.
2. Ice Pillow Group—graft positioned on a custom gauze-based ice pillow (Figure 1). This pillow was subsequently placed into the fluid-filled basin.

### 2.2. Ice Pillow Construction and Simulated Rewarming Model

To simulate the body cavity temperature, an isolation bag was placed over a metallic basin with water maintained at 30–32 °C, which has previously been shown to approximate the surface temperature of the body cavity during kidney transplantation [10].

The ice pillow was constructed by placing approximately 100 mL of crushed sterile ice into a laparotomy sponge, folded to create a flat pad approximately 12 cm × 6 cm and 4 cm thick. The gauze was secured by applying large surgical clips to the edges to keep the ice within the folded sponge. A new ice pillow was prepared for each experiment. The device was designed to provide cooling to one surface of the kidney only (Figure 1).

### 2.3. Temperature Measurement and Monitoring

Renal temperature assessment was performed using complementary core and surface thermometry techniques.

#### 2.3.1. Core Temperature Measurement

Core renal temperature was continuously monitored using a 12 Fr thermocouple probe inserted in atraumatic fashion through the ureter into the renal pelvis. The probe was connected to a Philips IntelliVue multiparameter monitoring system (2024 model) (Koninklijke Philips Electronics, Amsterdam, the Netherlands), allowing continuous real-time temperature acquisition. The thermocouple probe was calibrated against a NIST-traceable reference thermometer in an ice-water bath (0 °C) and warm water (37 °C) before each experiment.

#### 2.3.2. Surface Temperature Measurement

Surface renal temperature was measured using a non-contact CEM DT-8839 dual-laser infrared thermometer (Shenzhen Everbest Machinery Industry Co., Ltd., Shenzhen, China). Measurements were obtained with the device positioned approximately 10–15 cm perpendicular to the renal capsule to ensure consistent acquisition geometry and minimize measurement variability. The thermometer was factory calibrated, with a specified measurement accuracy of ±1.5% of the measured value or ±2.0 °C. The validity of infrared surface thermometry as a surrogate for core renal temperature has been previously demonstrated by our group in a porcine kidney model, where surface and core temperature measurements showed excellent correlation [16].

### 2.4. Experimental Phases and Data Collection

Temperature measurements were recorded at standardized experimental phases:

1. Baseline Phase: immediately following removal of the graft from hypothermic storage.
2. Rewarming Phase: serial temperature measurements obtained at one-minute intervals for 40 min under simulated implantation conditions.

The 40-min observation period was selected to approximate the duration of vascular anastomosis encountered during kidney transplantation procedures.

### 2.5. Outcome Measures

The primary outcome measure was the rate of renal rewarming (°C/min) during the simulated second warm ischemic period. Secondary outcome measures included:

- Mean renal temperature at predefined time intervals,
- Final recorded renal temperature,
- Temporal rewarming profile,
- Agreement between core and surface temperature measurements,
- Correlation between thermal measurement modalities.

### 2.6. Statistical Analysis

Continuous variables were summarized as mean ± standard deviation (SD). Prior to inferential testing, normality of paired differences was assessed using the Shapiro–Wilk test. All analyses accounted for the donor-paired study design, in which one kidney from each donor pair was assigned to Ice Pillow support and the contralateral kidney served as Control.

Rewarming kinetics were assessed by estimating an average rewarming slope for each kidney using linear regression of internal temperature over time; donor-paired slope differences were then calculated as Control minus Ice Pillow and compared using a paired *t*-test after assessment of normality of paired differences.

Longitudinal internal temperature trajectories were analyzed using a linear mixed-effects model to account for repeated temperature measurements within kidneys. The model was specified as:

Temperatureij = β_0_ + β_1_(Group) + β_2_(Timec) + β_3_(Timec^2^) + β_4_(Group × Timec) + β_5_(Group × Timec^2^) + β_6_(Donor Pair) + uj + εij

where uj represents the kidney-level random intercept and εij the residual error. Time was centered at the midpoint of the observation period (20 min). The model was fitted using restricted maximum likelihood (REML) estimation via the lme4 package in R.

The model included treatment group, centered time, centered time^2^, treatment-by-time interactions, and donor pair, with kidney-level random effects for repeated measurements. Model-estimated Control minus Ice Pillow temperature differences with 95% confidence intervals were calculated at predefined time points.

Agreement between core/internal and surface/external temperature measurements was evaluated using Bland–Altman analysis, with measurement difference defined as surface minus core temperature. Bias and 95% limits of agreement were reported overall and by kidney.

Statistical significance was defined as a two-tailed *p*-value of 0.05.

All statistical analyses were performed using R (Version 4.5.0; R Foundation for Statistical Computing, Vienna, Austria).

## 3. Results

A total of twelve human renal allografts (six donor pairs) were included in the analysis during simulated rewarming. Within each donor pair, kidneys were randomly assigned to either the Ice Pillow group (n = 6) or the Control group (n = 6) to simulate intraoperative handling during vascular anastomosis.

### 3.1. Rewarming Kinetics

**Figure 2:**
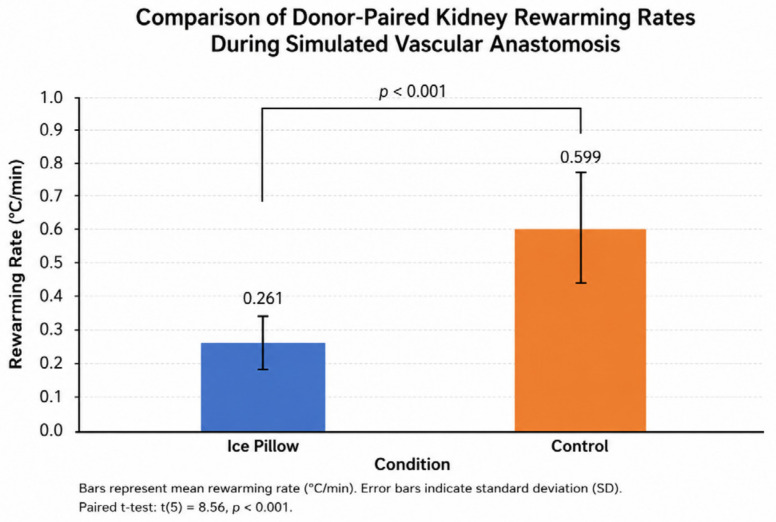
Comparison of donor-paired renal rewarming rates during simulated vascular anastomosis. Bars represent mean rewarming slope (°C/min), and error bars indicate standard deviation. Rewarming slopes were compared using a paired *t*-test.

Kidneys supported with the Ice Pillow demonstrated substantially slower rewarming kinetics than donor-paired Control kidneys (Table 1). For each kidney, the average rewarming slope was calculated using linear regression of internal kidney temperature over time, expressed as °C/min. Slopes were then compared within donor pairs by calculating the difference between the Control and Ice Pillow kidney from the same donor. The mean rewarming slope was 0.599 ± 0.066 °C/min for Control kidneys compared with 0.261 ± 0.057 °C/min for Ice Pillow kidneys, representing a 56.4% reduction in rewarming rate with Ice Pillow support. The mean donor-paired slope difference was 0.338 °C/min (95% CI, 0.237–0.440 °C/min). Normality of paired slope differences was confirmed using the Shapiro–Wilk test, and paired *t*-test analysis demonstrated significantly faster rewarming in Control kidneys than in their matched Ice Pillow counterparts (t(5) = 8.56, *p* < 0.001). All six donor pairs demonstrated faster rewarming under the Control condition (Figure 2).

**Figure 3:**
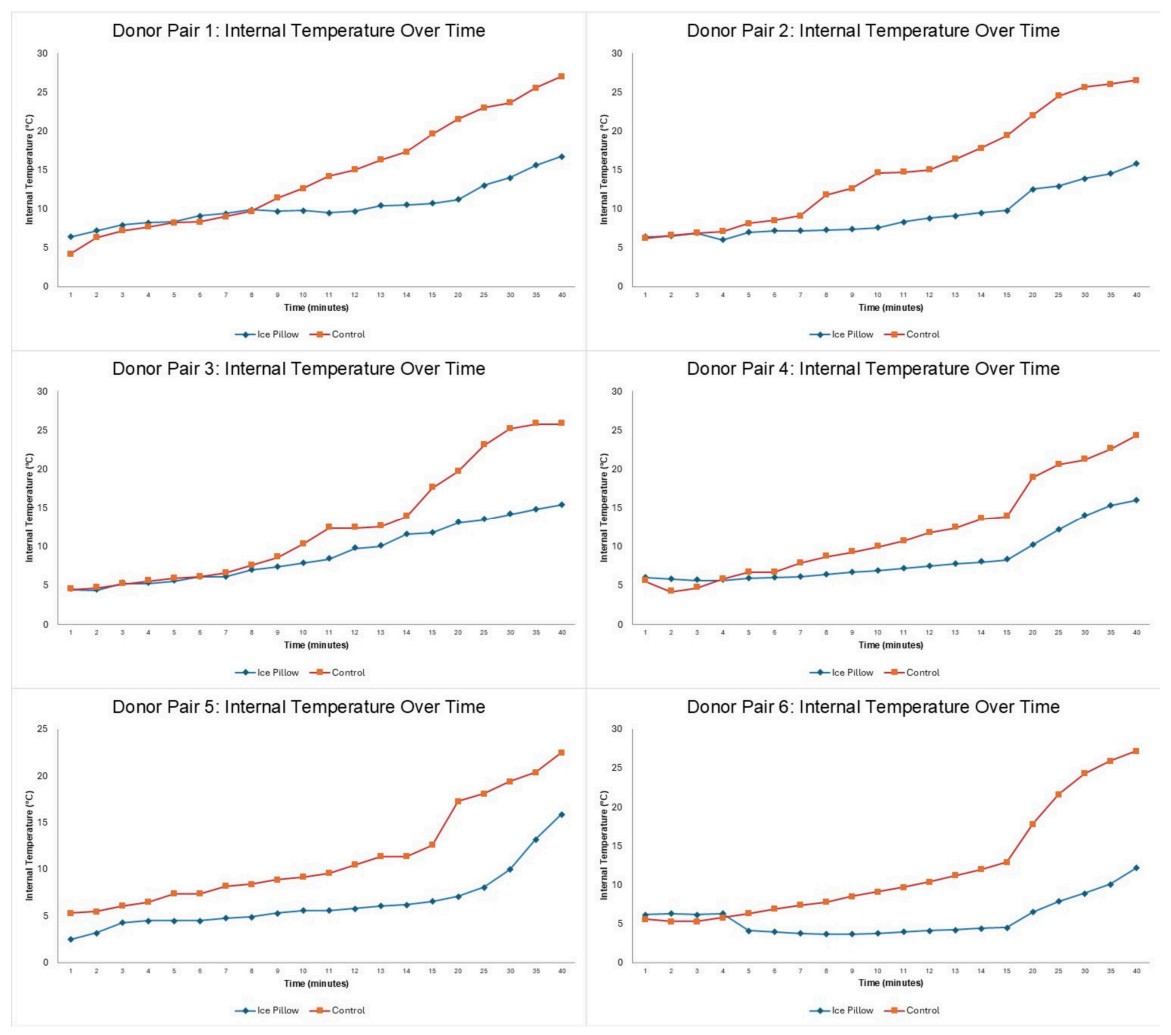
Internal renal temperature profiles for the six donor pairs during simulated vascular anastomosis. Within each donor pair, one kidney received Ice Pillow support (blue) and the contralateral kidney served as Control (orange). Internal temperature was recorded at one-minute intervals over 40 min.

Inspection of individual donor-pair temperature profiles demonstrated a consistent reduction in rewarming across all six donor pairs, with kidneys supported by the Ice Pillow maintaining lower internal temperatures throughout the 40-min observation period (Figure 3).

### 3.2. Longitudinal Internal Temperature Trajectories

Internal renal temperature trajectories differed significantly between Control and Ice Pillow kidneys over the 40-min simulated rewarming period. Because temperature measurements were repeatedly obtained from the same kidneys over time, longitudinal data were analyzed using a linear mixed-effects model. The model included treatment group, time, time^2^, treatment-by-time interactions, and donor pair, with kidney-level random effects to account for repeated measurements within each kidney. A quadratic time structure was selected because it provided substantially better fit than a linear-only time model, with lower Akaike information criterion values and significant improvement on likelihood-ratio testing (AIC 906.9 vs. 1028.1; χ^2^(2) = 125.1, *p* < 0.001).

**Figure 4:**
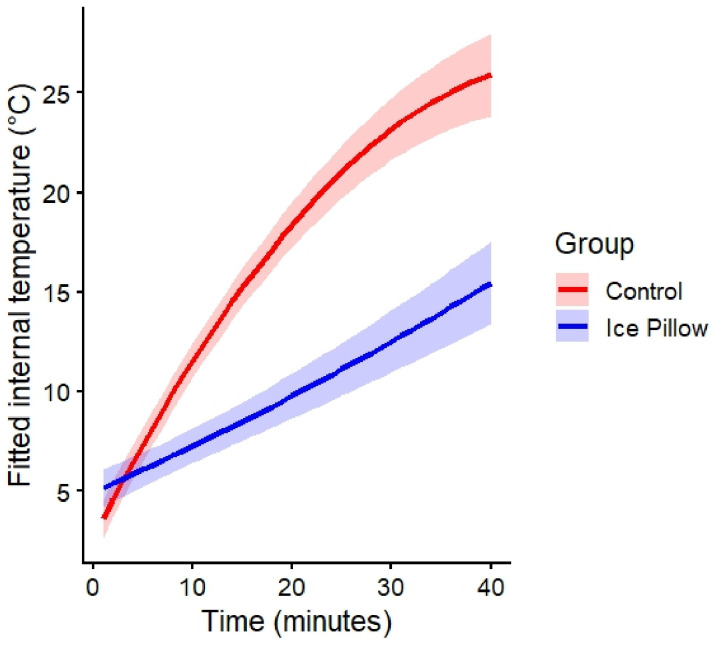
Model-fitted internal renal temperature trajectories derived from the quadratic linear mixed-effects model. Solid lines represent estimated mean temperatures and shaded bands indicate 95% confidence intervals.

Model-based contrasts demonstrated progressive thermal separation between groups, with Control kidneys becoming increasingly warmer than Ice Pillow kidneys over time (Table 2). At 10 min, Control kidneys were estimated to be 4.28 °C warmer than Ice Pillow kidneys (95% CI, 3.35–5.22; *p* < 0.001); this difference increased to 8.61 °C at 20 min (95% CI, 7.31–9.90; *p* < 0.001), 10.66 °C at 30 min (95% CI, 8.83–12.48; *p* < 0.001), and 10.44 °C at 40 min (95% CI, 7.88–12.99; *p* < 0.001). These findings indicate that Ice Pillow support significantly altered the longitudinal rewarming trajectory, resulting in sustained attenuation of internal renal temperature rise throughout the simulated second warm ischemic period (Figure 4).

At 40 min, the mean observed internal temperature was 15.28 °C in Ice Pillow kidneys compared with 24.31 °C in Control kidneys, placing Ice Pillow kidneys near the 15–18 °C metabolic threshold identified in the prior literature while Control kidneys had warmed substantially above it.

### 3.3. Core–Surface Temperature Agreement

**Figure 5:**
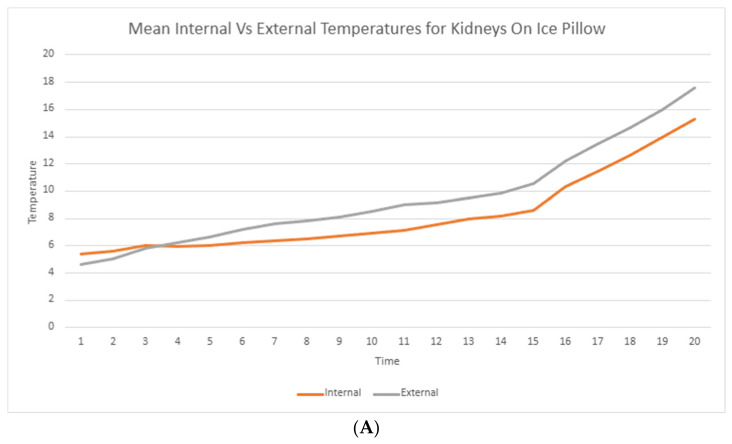

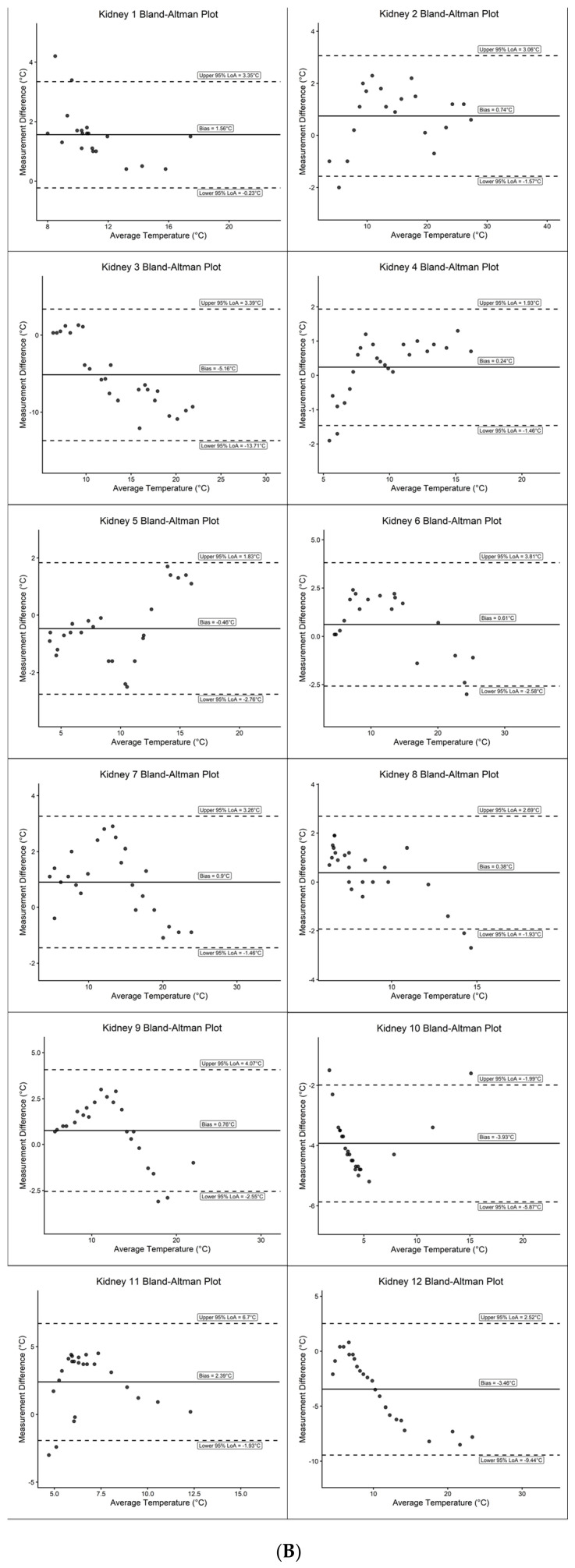
(**A**) Mean internal (orange) and external (gray) temperatures of Ice Pillow kidneys over time. Wilcoxon signed-rank test: W = 201, *p* < 0.001. (**B**) Bland–Altman plots showing agreement between surface and core renal temperature measurements for each of the 12 kidneys. The solid line represents the mean bias, and the dashed lines indicate the 95% limits of agreement.

Agreement between core and surface temperature measurements was evaluated using Bland–Altman analysis, with measurement difference defined as surface temperature minus core temperature. Across 276 paired measurements from 12 kidneys, the mean bias was −0.51 °C, indicating that surface temperature was, on average, slightly lower than core temperature. The repeated-measures-adjusted 95% limits of agreement were −6.46 °C to 5.43 °C. Individual kidney-level Bland–Altman plots demonstrated that agreement varied by kidney, with mean biases ranging from −5.16 °C to 2.39 °C (Figure 5B). Among Ice Pillow kidneys specifically, both internal and external temperatures increased progressively throughout the observation period. A Wilcoxon signed-rank test performed on paired kidney-level summary measurements demonstrated a significant difference between external (Mdn = 8.8 °C) and internal (Mdn = 7.0 °C) temperatures (W = 201, *p* < 0.001, r = 0.89). Normality testing of paired differences indicated a violation of normality (Shapiro–Wilk *p* = 0.0096), supporting the use of a nonparametric test (Figure 5A).

Overall, these findings suggest that surface thermometry followed the general direction of core temperature change over time; however, the wide 95% limits of agreement (−6.46 to +5.43 °C) and substantial between-kidney variability in bias (range: −5.16 to +2.39 °C) indicate that surface measurements cannot reliably substitute for core thermometry at the individual-measurement level.

Collectively, these findings demonstrate that the Ice Pillow provides a reproducible and statistically significant thermal preservation effect during simulated second warm ischemic exposure, as evidenced by donor-paired slope analysis, longitudinal mixed-effects modeling, and quantitative assessment of measurement agreement.

## 4. Discussion

In this controlled experimental study, the Ice Pillow significantly reduced the rate of renal rewarming during simulated implantation conditions, with a 56.4% reduction in rewarming velocity. This effect translated into a potentially relevant ~9 °C thermal separation between groups by 40 min (15.28 °C vs. 24.31 °C; *p* < 0.001), demonstrating sustained preservation of renal hypothermia throughout the simulated second warm ischemic period. The clinical significance of this difference is yet to be determined.

### 4.1. Biological Significance of the Thermal Threshold

The observed thermal separation as found in this experiment may be biologically relevant. In this context, Hameed et al. identified a metabolic threshold of approximately 15–18 °C, above which the protective effects of renal hypothermia are progressively lost [8]. Furthermore, Kuipers et al. demonstrated that in living-donor kidney transplantation, more than 80% of grafts exceed the 15 °C threshold within 20 min of the second warm ischemic period under standard conditions [9]. Our results showed that at 40 min, kidneys preserved on the Ice Pillow remained close to the critical 15 °C threshold (15.28 °C), whereas control kidneys had warmed substantially above it (24.31 °C). Earlier physiologic studies by Krzywonos-Zawadzka et al. showed significant suppression of renal metabolic activity and ATP-dependent transport processes at temperatures below approximately 15 °C [3]. Kamińska et al. also showed the profound reduction in ischemic injury that occurs when warm ischemia is minimized during transplantation [14]. Organ metabolic activity increases exponentially with temperature, and even relatively small increases in graft temperature may substantially increase oxygen demand during ischemia [15,16]. Consequently, maintaining graft temperatures near or below this threshold during implantation may represent a biologically meaningful target, although this hypothesis requires confirmation through studies measuring actual tissue injury markers and clinical outcomes.

### 4.2. Contextualization with Existing Cooling Strategies

These findings are consistent with and build upon previous research on active thermal protection strategies during kidney transplantation. The systematic review by Andras et al. provides a useful framework for the application of Ice Pillow in transplantation [12]. Simple instillation of cold serum is insufficient, with grafts reaching up to 33 °C at the end of warm ischemia [12]. Gauze jackets filled with ice slush, the most commonly reported technique, maintained graft temperatures up to 20.3 °C at the end of the warm ischemic period. However, concerns remain regarding inhomogeneous parenchymal cooling and the risk of secondary ileus. Novel continuous-flow devices maintain temperatures below 20 °C throughout anastomosis but require specialized equipment and additional operative setup [12,17]. The Ice Pillow achieved a final temperature of 15.28 °C at 40 min, which falls within the range reported for other cooling techniques, though direct comparison is limited by differences in study design (ex vivo vs. in vivo), sample size, and measurement methods.

Ide et al. demonstrated that a thermal barrier bag maintained a median graft surface temperature of 17.7 °C even with prolonged anastomosis times (median 53 min) performed by young transplant fellows [13]. Kamińska et al. showed that kidney surface cooling with the ice-bag technique significantly reduced the incidence of delayed graft function and/or acute rejection, while improving early glomerular filtration rate [14]. Zhang et al. reported that a continuous renal surface cooling technique during robotic-assisted kidney transplantation maintained significantly lower graft temperatures compared with the conventional ice slush method (15.42 ± 0.88 °C vs. 21.74 ± 2.53 °C at 20 min; *p* = 0.001), with corresponding improvements in early post-transplant creatinine levels [7]. These studies collectively support the principle that active intraoperative temperature management during vascular anastomosis can improve graft outcomes, and the Ice Pillow represents a practical, low-cost approach to achieving this goal.

### 4.3. Relevance of Rewarming Kinetics

The observed divergence in thermal trajectories between groups is particularly important because temperature kinetics may be more biologically relevant than isolated temperature measurements at fixed timepoints. Previous studies have shown that even modest increases in graft temperature during implantation may accelerate endothelial dysfunction, tubular injury, free radical generation, and reperfusion-associated oxidative injury [4,5,9,18]. Tennankore et al. demonstrated that warm ischemia time is associated with graft failure and mortality in a dose-dependent fashion, with each incremental increase in warm ischemia time conferring progressively higher hazard ratios for the composite outcome of death and graft failure [19]. Although this study did not measure biomarkers of cellular injury, the observed reduction in rewarming rate is consistent with the hypothesis that slowing the rate of rewarming may help preserve cellular homeostasis during vascular anastomosis, especially in technically complex transplant procedures or cases with prolonged implantation times [10]. These findings are also consistent with previous work comparing controlled versus passive rewarming strategies during renal allograft implantation, where temperature trajectory rather than isolated endpoint measurements appeared to be the more meaningful determinant of thermal protection [10].

### 4.4. Surgical Ergonomics and Practical Considerations

Unlike many conventional cooling adjuncts, the Ice Pillow was specifically designed to provide focused cooling while minimizing interference with surgical exposure. Traditional ice slush or blanket techniques can obscure visualization of the renal hilum, hinder vascular suturing, and increase operative complexity within the confined recipient pelvis [11]. In contrast, the low-profile design of the Ice Pillow allows continuous exposure of the hilar structures while preserving localized cooling of the dependent graft surface. Owing to its low cost, ease of preparation, and ready availability, this technique may offer a practical approach for maintaining renal hypothermia during implantation, particularly in resource-limited settings where continuous-flow cooling systems or specialized thermal barrier bags are not accessible.

### 4.5. Surface Thermometry as an Intraoperative Monitoring Tool

An additional important finding was the moderate concordance between internal and external temperature measurements throughout the rewarming interval. Surface temperature measurements correlated strongly with core temperatures, suggesting that infrared thermometry may provide a practical non-invasive method for intraoperative monitoring of graft temperature [16]. This finding supports further evaluation of surface thermometry within clinical renal transplantation protocols, as real-time temperature feedback could enable surgeons to make informed decisions about the need for additional cooling measures during prolonged anastomoses.

### 4.6. Limitations

Several limitations should be acknowledged. First, this study was designed to evaluate thermal behavior rather than direct biologic injury; biomarkers of ischemia–reperfusion injury, tissue ATP content, oxidative stress markers, and histologic assessment were not evaluated. Second, as an ex vivo experimental study, it was not designed to evaluate clinical endpoints such as delayed graft function, serum creatinine recovery, graft survival, or histologic ischemia–reperfusion injury; consequently, direct conclusions regarding clinical efficacy cannot yet be made. Third, the absence of systemic circulation, immune activation pathways, and recipient physiologic responses limits extrapolation to in vivo transplantation conditions. Fourth, all grafts had previously undergone ex vivo machine perfusion studies prior to re-equilibration. Although this may have altered baseline tissue characteristics, all kidneys were subsequently re-equilibrated to a standardized starting temperature of 4–6 °C. Fifth, although the study included twelve human renal allografts with paired randomization from six donors, the sample size remains modest and may not fully account for inter-organ variability. Finally, the warm basin-based control model maintained at 30–32 °C may not completely replicate the thermal characteristics of the recipient pelvic cavity, where ambient tissue temperatures may be higher and where blood flow from surrounding tissues contributes additional heat transfer.

Despite these limitations, the present study adds to the growing body of evidence supporting active management of intraoperative graft temperature during kidney transplantation. The Ice Pillow represents a simple, inexpensive, and surgically unobtrusive cooling adjunct capable of significantly attenuating renal rewarming during simulated vascular anastomosis. Its ease of preparation, low cost, and minimal interference with operative exposure may facilitate integration into both open and minimally invasive transplant workflows. Further prospective clinical studies are warranted to determine whether the observed reduction in rewarming kinetics translates into improved graft function and post-transplant outcomes.

## 5. Conclusions

In this ex vivo model using human renal allografts, the Ice Pillow reduced the rate of renal rewarming by more than 50% compared with unassisted controls and maintained core temperatures closer to the 15–18 °C threshold over a 40-min simulated anastomosis period. These findings are limited to thermal performance in a laboratory setting and do not establish clinical efficacy. Prospective clinical studies are warranted to determine whether this thermal effect translates into reduced delayed graft function and improved graft outcomes.

## Figures and Tables

**Table 1:** Analysis: Donor-paired rewarming slope analysis using a paired *t*-test.

Parameter	Result
Control slope ± SD (°C/min)	0.599 ± 0.066
Ice Pillow slope ± SD (°C/min)	0.261 ± 0.057
Mean paired difference (°C/min)	0.338
95% CI	0.237 to 0.440
Paired *t*-test	t(5) = 8.56
*p*-value	*p* < 0.001
Percent reduction	56.40%

**Table 2:** Model-estimated temperature differences between Control and Ice Pillow kidneys at predefined time points. Differences were derived from a quadratic linear mixed-effects model with treatment group, centered time, centered time^2^, treatment-by-time interactions, donor pair, and kidney-level random effects. Positive values indicate higher temperatures in Control kidneys.

Time (Minutes)	Model-Estimated Control − Ice Pillow Difference, °C	95% CI	*p*-Value
1	−1.55	−2.67 to −0.42	0.0069
5	1.27	0.32 to 2.22	0.0086
10	4.28	3.35 to 5.22	<0.001
20	8.61	7.31 to 9.90	<0.001
30	10.66	8.83 to 12.48	<0.001
40	10.44	7.88 to 12.99	<0.001

## Data Availability

The data supporting the findings of this study are available from the corresponding author upon reasonable request.
